# Early Detection of Hypothermic Neuroprotection Using T2-Weighted Magnetic Resonance Imaging in a Mouse Model of Hypoxic Ischemic Encephalopathy

**DOI:** 10.3389/fneur.2018.00304

**Published:** 2018-05-08

**Authors:** Sydney E. Doman, Akanksha Girish, Christina L. Nemeth, Gabrielle T. Drummond, Patrice Carr, Maxine S. Garcia, Michael V. Johnston, Sujatha Kannan, Ali Fatemi, Jiangyang Zhang, Mary Ann Wilson

**Affiliations:** ^1^Hugo W. Moser Research Institute at Kennedy Krieger, Baltimore, MD, United States; ^2^Department of Neurology, The Johns Hopkins University School of Medicine, Baltimore, MD, United States; ^3^Anesthesiology and Critical Care Medicine, The Johns Hopkins University School of Medicine, Baltimore, MD, United States; ^4^Department of Radiology, New York University School of Medicine, New York, NY, United States; ^5^Department of Neuroscience, The Johns Hopkins University School of Medicine, Baltimore, MD, United States

**Keywords:** hypothermia, neuroprotection, hypoxic-ischemic, neonatal encephalopathy, magnetic resonance imaging

## Abstract

Perinatal hypoxic-ischemic encephalopathy (HIE) can lead to neurodevelopmental disorders, including cerebral palsy. Standard care for neonatal HIE includes therapeutic hypothermia, which provides partial neuroprotection; magnetic resonance imaging (MRI) is often used to assess injury and predict outcome after HIE. Immature rodent models of HIE are used to evaluate mechanisms of injury and to examine the efficacy and mechanisms of neuroprotective interventions such as hypothermia. In this study, we first confirmed that, in the CD1 mouse model of perinatal HIE used for our research, MRI obtained 3 h after hypoxic ischemia (HI) could reliably assess initial brain injury and predict histopathological outcome. Mice were subjected to HI (unilateral carotid ligation followed by exposure to hypoxia) on postnatal day 7 and were imaged with T2-weighted MRI and diffusion-weighted MRI (DWI), 3 h after HI. Clearly defined regions of increased signal were comparable in T2 MRI and DWI, and we found a strong correlation between T2 MRI injury scores 3 h after HI and histopathological brain injury 7 days after HI, validating this method for evaluating initial injury in this model of HIE. The more efficient, higher resolution T2 MRI was used to score initial brain injury in subsequent studies. In mice treated with hypothermia, we found a significant reduction in T2 MRI injury scores 3 h after HI, compared to normothermic littermates. Early hypothermic neuroprotection was maintained 7 days after HI, in both T2 MRI injury scores and histopathology. In the normothermic group, T2 MRI injury scores 3 h after HI were comparable to those obtained 7 days after HI. However, in the hypothermic group, brain injury was significantly less 7 days after HI than at 3 h. Thus, early neuroprotective effects of hypothermia were enhanced by 7 days, which may reflect the additional 3 h of hypothermia after imaging or effects on later mechanisms of injury, such as delayed cell death and inflammation. Our results demonstrate that hypothermia has early neuroprotective effects in this model. These findings suggest that hypothermia has an impact on early mechanisms of excitotoxic injury and support initiation of hypothermic intervention as soon as possible after diagnosis of HIE.

## Introduction

Perinatal hypoxic-ischemic encephalopathy (HIE) is a childbirth complication most commonly induced by intrauterine asphyxia (1). Neonatal HIE can lead to a wide spectrum of neurodevelopmental disorders, most notably cerebral palsy (CP) (2). While CP is a non-progressive disorder, HIE injury is a dynamic process. Injury begins when cerebral blood flow and oxygen delivery to the brain are impaired, resulting in primary energy failure (3–5). Secondary energy failure occurs 6–48 h later, from a combination of oxidative stress, excitotoxicity, and inflammation (6, 7). The window between primary and secondary energy failure provides a critical period for intervention to mitigate or altogether prevent the effects of secondary energy failure (8). Early classification of HIE severity may allow more appropriate intervention, such as identification of patients who would benefit from more aggressive or pharmaceutical treatment (9).

Therapeutic hypothermia is the standard treatment for neonatal HIE, due to its proven efficacy in reducing infant mortality and brain injury by 25%, and in reducing the risk of CP by 34% (10). The timing and intensity of cooling during therapeutic hypothermia are critical in preserving neural function. In clinical practice, neonates begin hypothermia within 6 h of birth and are typically maintained at 33.5 ± 0.5°C for 72 h (11). Cooling below 33.5°C provided no further neuroprotection in a preclinical study of rat pups (12), and this has now been confirmed in a clinical trial (11). Moreover, studies across a variety of animal models have shown a negative correlation between overcooling and neuroprotection. Hypothermia performed at 8.5°C below normothermia in piglets increased brain cell death (13), and overcooling in the rat model to 18°C provided no neuroprotection and had a negative impact on feeding ability (12). We have validated a model of hypothermic neuroprotection in mice subjected to hypoxic ischemic insult on postnatal day 7 (P7) that replicates the neuroprotective effects of mild hypothermia in term human infants (14).

Magnetic resonance imaging (MRI) has numerous benefits as a non-invasive tool to assess brain injury in neonates. There is debate over the optimal timing for MRI in order to reliably assess and predict outcomes of brain injury. A report from the American College of Obstetricians and Gynecologists has affirmed that an initial diagnostic MRI obtained between 24 and 96 h shows distinguishable patterns of injury in myelinated regions of the brain, while scans obtained after 10 days of life offer the most conclusive evidence for the degree of cerebral injury (15). In infants with HIE, early MRI (≤6 days of age, typically at the end of therapeutic hypothermia) has been shown to be sensitive and specific for predicting neurodevelopmental outcome, with similar performance to MRI performed after 7 days of age (16). Furthermore, a study of newborns with HIE and MRI studies performed a median of 6 days after birth found that the prognostic value of MRIs was unaffected by therapeutic hypothermia (17). MRI is typically obtained at the end of hypothermia, but a recent study evaluated imaging carried out during hypothermia. Although MRI performed on day 1 of life tended to underestimate brain injury, brain injury examined using DWI at day 2–3 of life fully corresponded with assessments carried out using T1/T2-weighted imaging at day 10 of life (18). Establishing reliable MRI at earlier time points may facilitate the differentiation of mild, moderate, and severe cases of HI insult, which could have a decisive impact on treatment.

Immature rodent models of HIE, in which carotid artery ligation is followed by a period of hypoxia, produce a distribution of brain injury that resembles the injury observed in asphyxiated term human infants (19, 20). The timing of brain injury in these models differs from that reported in human infants, with a more rapid progression of injury detected in mouse models of stroke and HIE. A number of studies have shown that injury can be reliably assessed with diffusion-weighted or T2 MRI, 3–24 h after the initial insult in various rat and mouse models of neonatal brain injury (21–27). In this study, we first sought an efficient method for assessing initial injury in our mouse model of perinatal HIE, for use in studies of hypothermic neuroprotection and for later use in developing complementary drug therapies. We compared T2-weighted and diffusion-weighted MRI obtained 3 h after HI for predicting brain histopathological outcome at 7 days in this model and found that they detected a very similar distribution and extent of injury that corresponded well with histopathological outcome at 7 days. We then applied the more efficient T2-weighted MRI at 3 h after HI to characterize initial injury in mice treated with hypothermia and found differences between the treatment groups at this early time point. The final phase of this study examined whether hypothermic neuroprotection that was detected with MRI 3 h after HI was maintained at a longer survival, using both T2 MRI and histopathology 7 days after HI. Our results confirm the prognostic value of early T2-weighted MRI as a predictor of HI brain injury in this mouse model, in both untreated mice and in mice treated with therapeutic hypothermia. These data also reveal that neuroprotective effects of hypothermic treatment can be detected as early as 3 h after initiating hypothermia in this model of HIE. These findings suggest that hypothermia can ameliorate very early mechanisms of brain injury in HIE, which may have important implications for the development of complementary therapies for use with hypothermia.

## Materials and Methods

### Experimental Design

**Figure 1:**
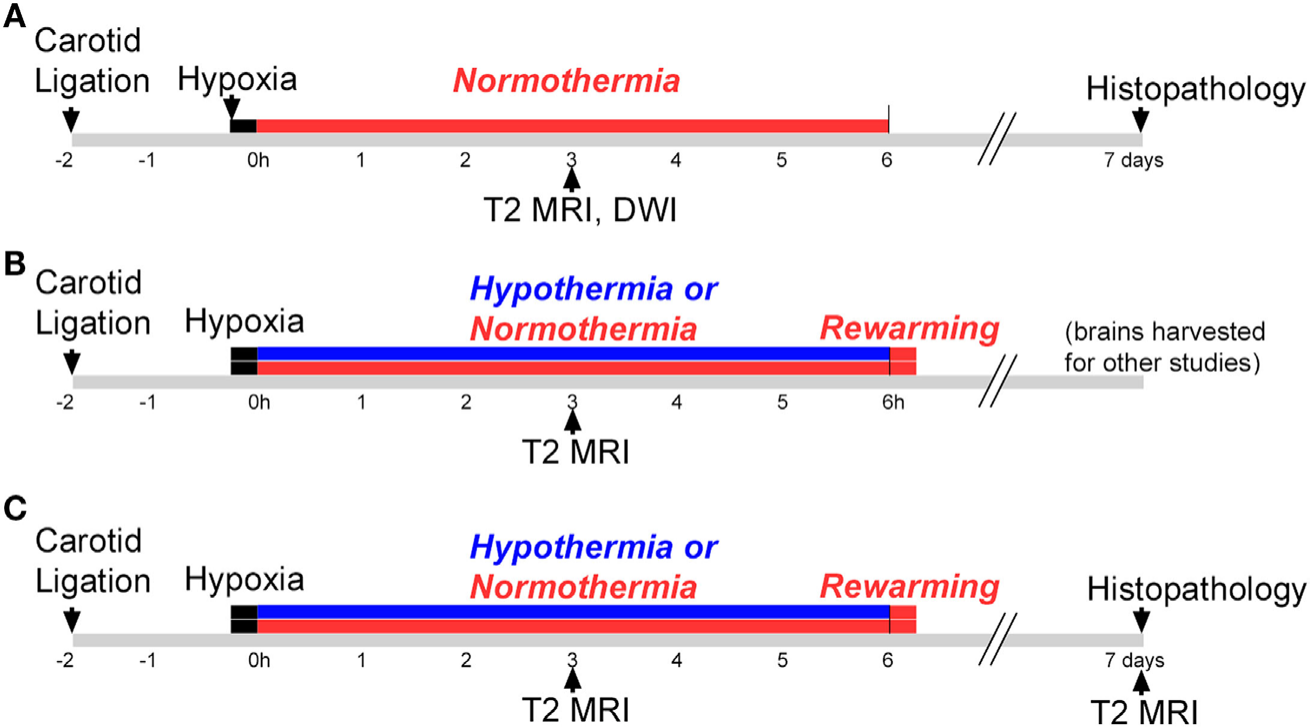
Experimental timelines. Experiments were conducted in three phases. **(A)** Phase 1: mice were subjected to hypoxic ischemia (HI) on postnatal day 7 (P7), followed by a 6 h period of normothermia. T2-weighted magnetic resonance imaging (MRI) and diffusion-weighted MRI (DWI) were carried out 3 h after HI. Brain injury was scored in T2 MRI using a qualitative scale. Mice were euthanized and histopathology was examined 7 days after HI, on P14. **(B)** Phase 2: mice were subjected to HI on P7, followed by a 6 h period of hypothermia or normothermia (brains were harvested for other studies not shown here). The validated T2 MRI score was determined at 3 h. **(C)** Phase 3: mice were subjected to HI on P7, followed by a 6 h period of normothermia or hypothermia. T2-weighted MRI was obtained and scored 3 h and 7 days after HI, and brain injury was examined in histological sections 7 days after HI.

This study consisted of three phases, illustrated in Figure 1. All phases used a P7 mouse model of HIE (28), and Phases 2 and 3 evaluated hypothermic neuroprotection, carried out as described previously (14). Phase 1 compared early T2-weighted and diffusion-weighted MRI for prediction of histopathologic brain injury examined at P7 (Figure 1A). Phase 2 applied the validated T2-weighted MRI scale in mice treated with hypothermia and in normothermic littermates, to characterize initial injury for a separate study (Figure 1B). Because Phase 2 showed striking differences in MRI injury scores between normothermic and hypothermic groups as early as 3 h after HI, Phase 3 was conducted to determine whether hypothermic neuroprotection demonstrated using T2-weighted MRI scores 3 h after HI persisted at a longer survival time, using both T2-weighted MRI and histopathologic brain injury examined 7 days after HI (Figure 1C).

### Animal Model of HIE

All animal procedures were carried out in accordance with the National Research Council Guide for the Care and Use of Experimental Animals (29) and were approved by the Institutional Animal Care and Use Committee of Johns Hopkins University. Lactating CD1 female mice with litters consisting of five male and five female pups were obtained on P6 (Charles River Laboratories) and housed in barrier facilities with a 14 h light/10 h dark schedule and standard mouse chow and water *ad libitum*. The following day, pups were subjected to HI as described previously (14). Briefly, under isoflurane anesthesia, the right common carotid artery was permanently ligated. Pups recovered in an incubator at 36.5°C for approximately 90 min and were then placed in hypoxic chambers (10% O2, balance N2) for 20 min at 36.5°C. Pups were randomly assigned to hypothermic treatment (6 h at 33.5°C) or a control period of normothermia (6 h at 36.5°C), as described previously (14). The hypothermic temperature was selected to correspond to that used clinically for HIE, and the length of hypothermic therapy was estimated as the longest period that pups could be maintained in incubator chambers, separated from their dam. We have shown previously that these conditions result in moderate neuroprotection [(14), supplemental Figure S2]. The number of subjects in each treatment group for each phase is shown in Table 1. Hypothermic and normothermic body temperatures (average of readings at 30 min and 6 h) were 33.6 ± 0.4°C and 36.9 ± 0.3°C, respectively. All pups were then kept at 36.5°C for 15 min before returning to the dam.

**Table 1:** Distribution of surviving pups by treatment group in each phase and mortality.

Phase	Normothermia		Hypothermia		Mortality	
	Male	Female	Male	Female	Total	*n*
1	23	20			7 (14%)	43
2	14	17	15	16	2 (3%)^a^	62
3	3	4	3	5	1 (6.25%)	15

*^a^Pups were not kept until the 7-day time-point; mortality rate reflects that observed after 6 h treatment*.

### *In Vivo* MRI

Magnetic resonance imaging was carried out 3 h after HI in all pups across all phases and again 7 days after HI in Phase 3 pups. All MRI studies were performed in the F.M. Kirby Research Center High-Field Preclinical MR Facility at the Kennedy Krieger Institute on a Bruker BioSpec horizontal 11.7 T MRI system. A custom holder was designed to permit scanning of mice in pairs, using a receive-only rat head phased array coil in combination with a quadrature volume coil for excitation. Anesthesia was induced with 2% isoflurane and maintained with 1.0 to 1.5% isoflurane in a mixture of O_2_ and air. After an initial localizer scan, T2-weighted images were acquired using the rapid acquisition with relaxation enhanced sequence and the following parameters: echo time (TE)/repetition time (TR) = 50/5,500 ms, 4 signal averages, echo train length = 8, field of view (FOV) = 23 mm × 14 mm, matrix size = 256 × 128, an in-plane resolution of 0.09 mm × 0.11 mm, 41 slices with a slice thickness of 0.5 mm that cover the entire brain, and a total imaging time of 5 min. Diffusion-weighted images were acquired using the diffusion weighted echo planar imaging sequence and the following parameters: TE/TR = 27/5,000 ms, 1 signal averages, 4 segments, FOV = 23 mm × 14 mm, matrix size = 174 × 96, an in-plane resolution of 0.13 mm × 0.15 mm, gradient duration/separation = 4/12 ms, 30 diffusion-encoding directions with a diffusion-weighting (b) of 800 s/mm^2^, 5 non-diffusion-weighted images, 20 slices with a slice thickness of 1 mm that cover the entire brain, and a total imaging time of 11 min. For studies in Phases 2 and 3, the bed and air temperatures in the MRI were adjusted to maintain normothermic or hypothermic body temperatures.

### Histopathology

To evaluate brain injury in histological sections 7 days after HI, mice were perfused with ice-cold phosphate-buffered saline. Brains were removed, immersion fixed in methacarn fixative (60% methanol, 30% chloroform, 10% glacial acetic acid) and embedded in paraffin. Brains were sectioned at 20 µm, and a 1 in 10 series of sections was stained with cresyl-violet.

### Histopathological Image Analysis

In each subject, a series of regularly spaced sections extending throughout the brain was imaged using a 1.25× objective on a Zeiss Axio Scope microscope. MCID Core 7.1 (Interfocus Imaging, LTD) was used to carry out a volumetric analysis of injury in the injured right hemisphere, compared with the uninjured contralateral left hemisphere. Brain injury was calculated as a percentage of the total contralateral volume, [(contralateral − ipsilateral/contralateral) × 100]. The contralateral hemisphere typically served as a reliable uninjured control. However, in very severely injured cases, injury in the contralateral hemisphere, typically in the cerebral cortex (cortex MRI score of 4), invalidated its use as a comparison control. A standard volume for the control hemisphere was calculated as the mean hemispheric volume of hemispheres in uninjured pups (MRI score of 0). Contralateral and ipsilateral hemispheric volumes did not differ significantly in the uninjured pups (paired *t*-test, *p* = 0.351), and thus both were used for an accurate control. This standard hemispheric volume was substituted for cases in which contralateral injury was observed and the contralateral volume was more than two SDs below the uninjured mean.

### MRI Analysis

**Figure 2:**
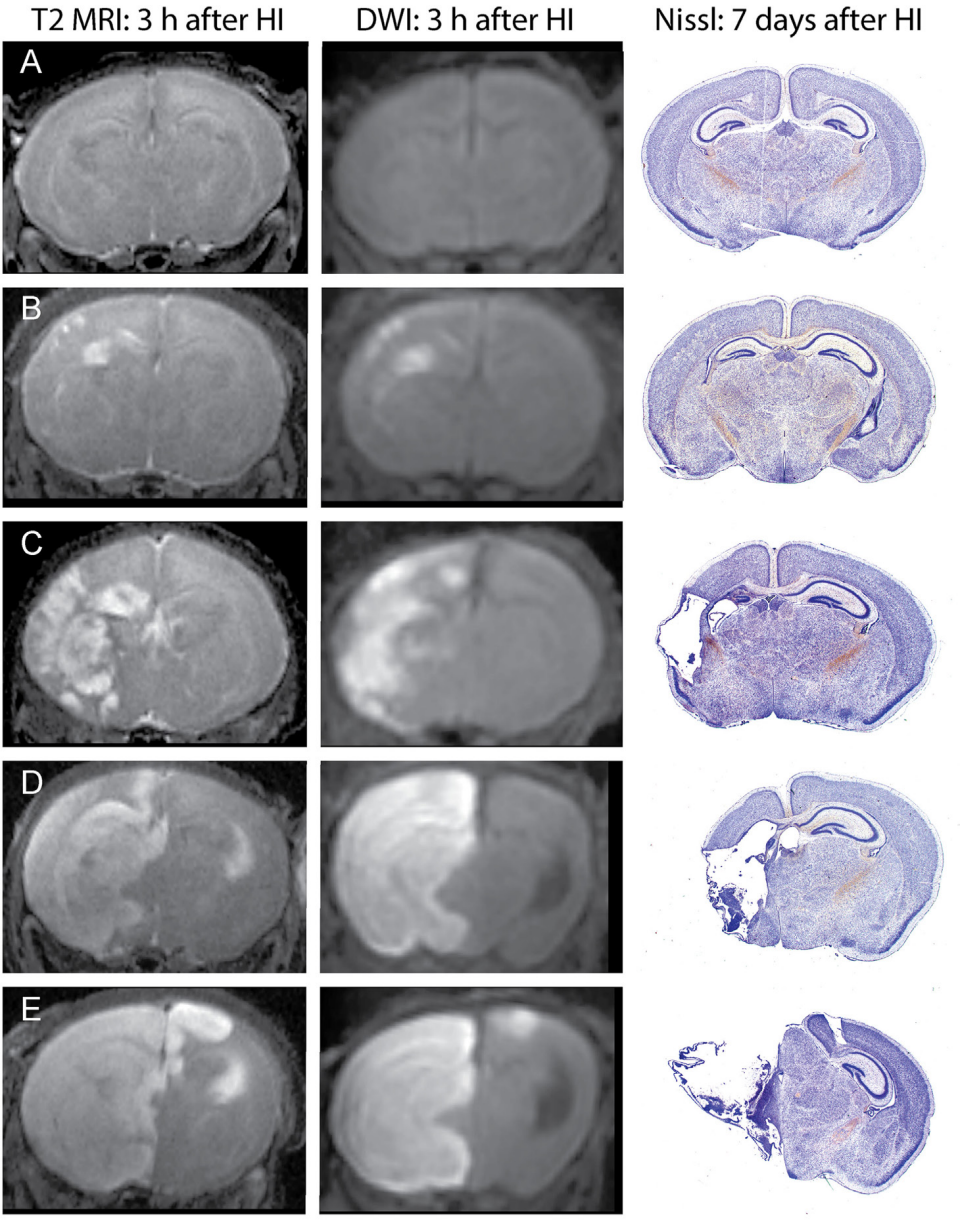
Phase 1: T2-weighted magnetic resonance imaging (MRI) and trace DWI obtained 3 h after hypoxic ischemia (HI), with corresponding histological sections demonstrating brain injury, 7 days after HI. Representative images show increased signal consistent with edema in T2-weighted MRIs and a similar pattern of injury in DWI, with corresponding brain injury observed at 7 days in cresyl-violet stained brain sections. Brain injury was scored in the T2-weighted images, with the following total injury scores in the examples shown: **(A)** no injury (MRI score of 0), **(B)** mild injury (MRI score 3.5), **(C)** moderate injury (MRI score 8), **(D)** severe unilateral injury (MRI score 12), **(E)** severe bilateral injury (MRI score 15).

Unlike in MRI of patients with ischemic stroke, which often show delayed increases in T2 signals by several hours after changes in DWI signals, in this mouse model, increased T2 signals can be readily detected in edema regions 3 h after HI. DWI data were processed using ParaVision 5.1 DTI tensor reconstruction to generate fractional anisotropy, tensor trace, intensity, and trace weighted images, which were compared with T2 MRI for evaluation of injury. Brain injury was most clearly delineated in the T2 MRI and trace DWI, shown in Figure 2. The injury was comparable using these methods, but T2 MRI provided a more detailed assessment of the extent and regional distribution of injury, with less time required for imaging, at higher resolution, than that required for DWI. (A lower resolution for DWI was necessitated by overall imaging/anesthesia time constraints and a voxel size permitting adequate diffusion signals.) The higher resolution T2 MRI were obtained using much shorter imaging times that limited the amount of additional anesthesia and minimized potential interference with thermal control. T2 MRI was thus selected for scoring and used exclusively in the later experiments. Each series of coronal T2 images extending throughout the forebrain was examined by two investigators who were unaware of treatment status. Cortex, hippocampus, striatum, and thalamus were scored using an ordinal scale similar to that used previously to score histopathology in this model (28) as follows: (0) no apparent injury; (1) mild injury, consisting of small hyperintense areas (<30% of the regional volume); (2) moderate injury, larger hyperintense areas (30–60% of total regional volume); (3) severe injury (>60% of the regional volume); or (4) severe with bilateral injury in that region. The regional scores were summed to obtain a total brain injury score, with a maximum possible score of 16. Representative samples are shown in Figure 2. No significant difference was found between the two investigator’s MRI scores (Wilcoxon signed-rank test, *p* = 0.48), and the average of their scores was used for analysis. A similar regional scoring model was used to evaluate brain injury 7 days after HI in Phase 3, when injury consisted of cystic infarction and atrophy: (0) no injury apparent; (1) mild injury (<30% regional volume loss); (2) moderate injury (30–60% regional volume loss); (3) severe injury (>60% regional volume loss); or (4) severe with bilateral injury in that region.

### Data Analysis

Statistical analyses were performed using IBM SPSS Statistics 24. Independent samples *t*-tests were used to analyze differences in volumetric brain injury across treatment groups. Paired *t*-tests were used to compare right and left uninjured hemispheres. Non-parametric tests were used for analysis of MRI scores: Spearman’s non-parametric correlation, related samples Wilcoxon signed-rank tests for comparison of MRI scores at 3 h and 7 days, independent samples Mann–Whitney *U*-tests for comparison of MRI scores between treatment groups. Differences were considered significant at *p* < 0.05.

## Results

### Phase 1: Validation of MRI Score at 3 h to Predict Brain Injury at 7 Days

**Figure 3:**
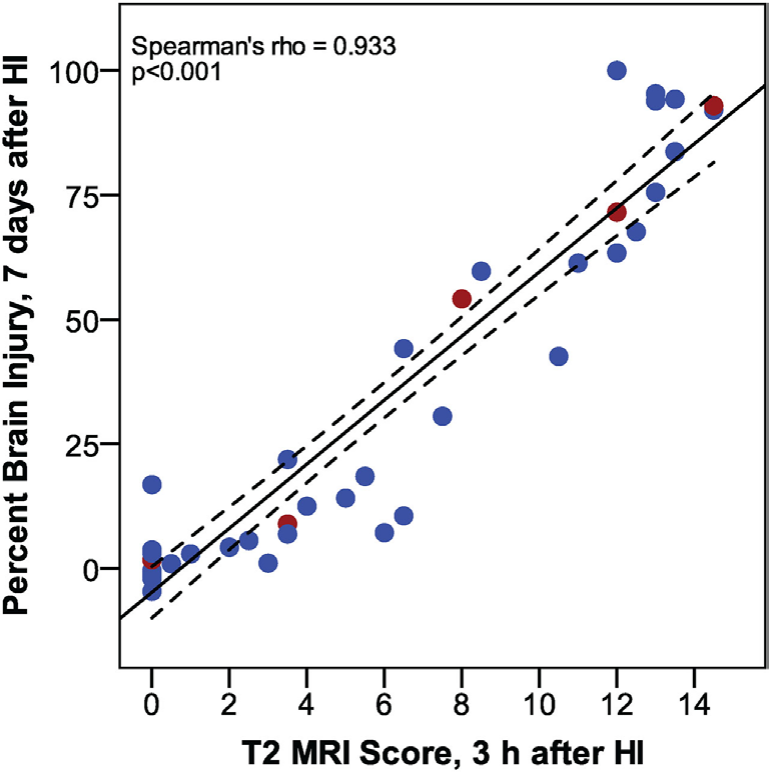
Phase 1: correlation between T2 magnetic resonance imaging scores, 3 h after hypoxic ischemia (HI), and percent brain injury measured in histological brain sections, 7 days after HI. (*n* = 43. Samples shown in Figure 2 are colored red.)

Increased T2 signals were readily detected in edema regions 3 h after HI (Figure 2), and T2 MRI and trace DWI showed a comparable extent and distribution of edema (Figure 2). T2 MRI provided a more detailed assessment of the extent and regional distribution of injury, with less time required for imaging, at higher resolution, than that required for DWI. T2 MRI scores at 3 h in normothermic pups were highly correlated with histopathological brain injury at 7 days (Figure 3, Spearman’s rho = 0.93, *p* < 0.001). Thus, in this murine model of neonatal HIE, very early T2-weighted MRI provides a reliable method for predicting the severity of brain injury exhibited 7 days after HI. Refer to Table 1 for number of animals used.

### Phase 2: Early Hypothermic Neuroprotection Detected by T2-Weighted MRI, 3 h After HI

**Figure 4:**
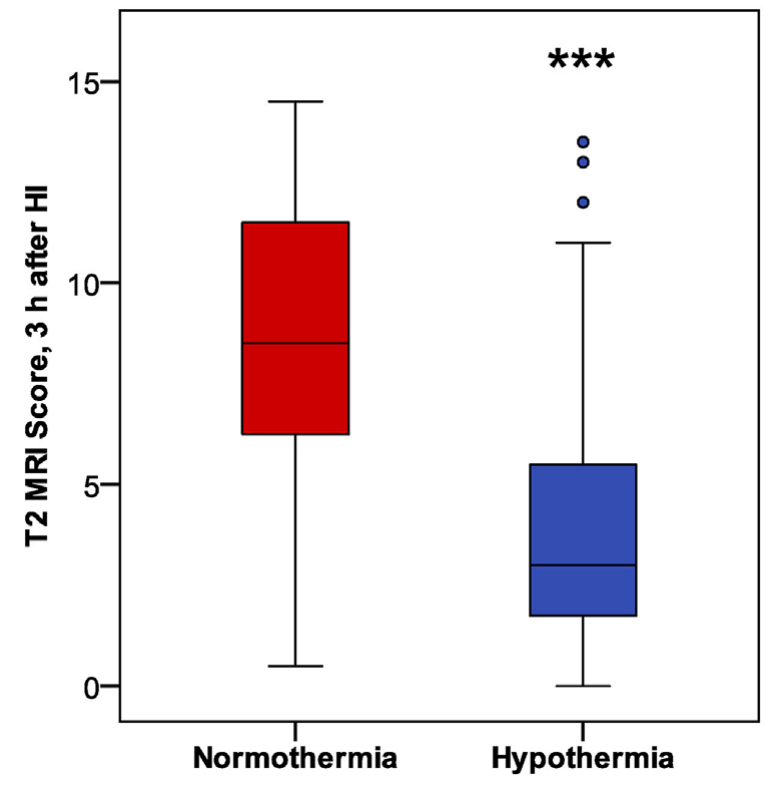
Phase 2: early detection of hypothermic neuroprotection in T2 magnetic resonance imaging, 3 h after HI (*n* = 31 in each treatment group; ***p < 0.001, Mann–Whitney *U*-test).

In mice subjected to HI followed by a 6 h period of hypothermia or normothermia, T2-weighted MRI was obtained 3 h after HI, to evaluate initial injury. As the MRI was obtained, a qualitative assessment was noted, distinguishing no injury, mild, moderate, and severe injury in these subjects, which revealed a difference in apparent injury between hypothermic and normothermic pups. The regional scoring method validated in our Phase 1 study was then applied, and we found a striking reduction in brain injury in the hypothermic group, after only 3 h of hypothermia (Figure 4). This study establishes a surprisingly early time point for the detection of hypothermic neuroprotection. Refer to Table 1 for number of animals used.

### Phase 3: Validation of Prognostic Value of Early T2-Weighted MRI and Confirmation of Sustained Hypothermic Neuroprotection at 7 Days

**Figure 5:**
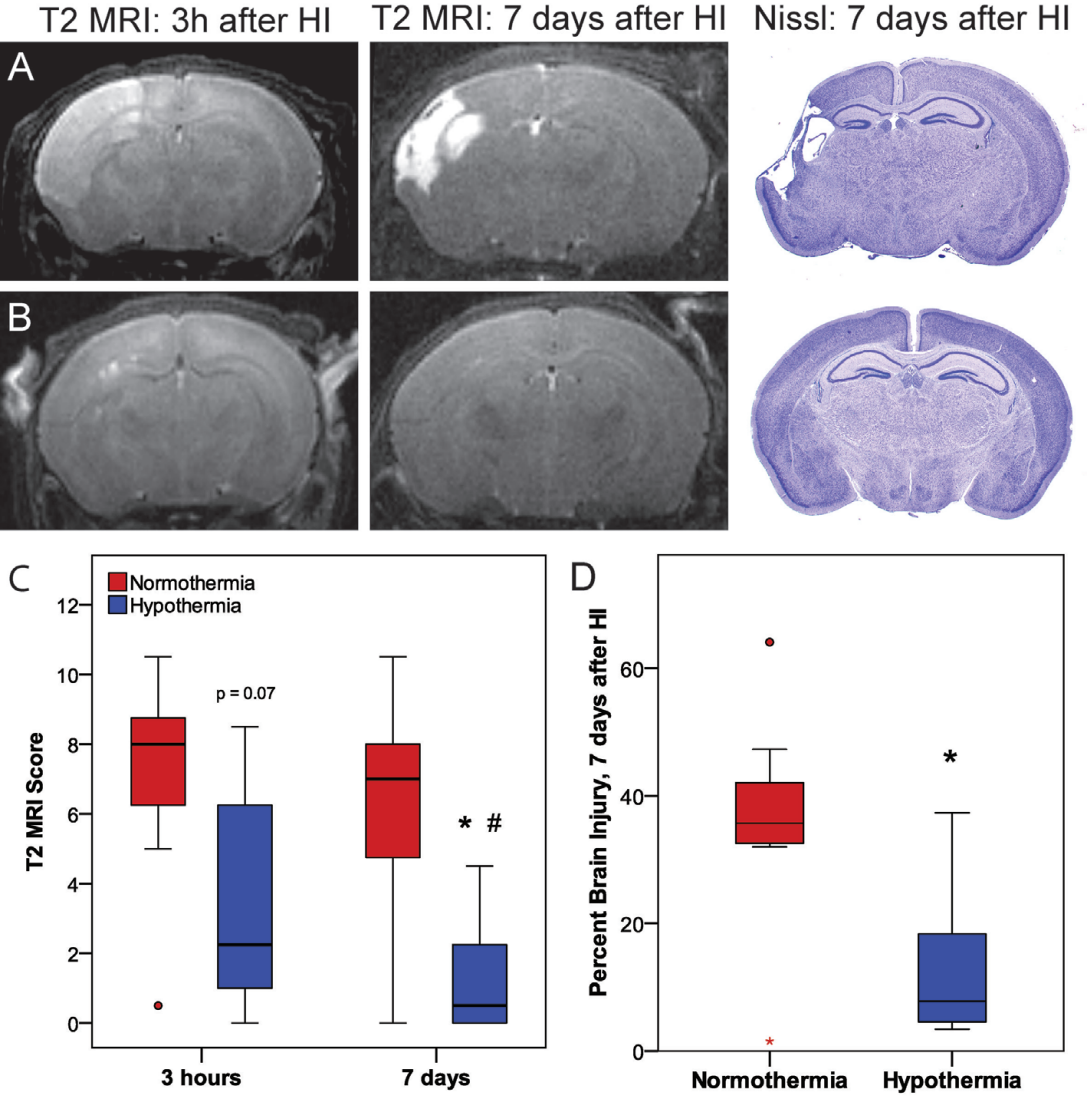
Phase 3: **(A,B)** representative T2 magnetic resonance imaging (MRI) obtained 3 h or 7 days after hypoxic ischemia (HI), with corresponding histopathology at 7 days in **(A)** normothermic or **(B)** hypothermic mice. **(C)** T2-weighted MRI scores at 3 h or 7 days after HI. With the smaller number of subjects examined in Phase 3 (*n* = 7 or 8 per treatment group), there was a trend for lower T2 MRI scores in the hypothermic group at 3 h compared to the normothermic group at 3 h (*p* = 0.072, Mann–Whitney *U*-test) and significantly lower T2 MRI scores in the hypothermic group at 7 days compared to the normothermic group at 7 days (*p < 0.05, Mann–Whitney *U*-test). In normothermic subjects, T2 MRI scores were comparable 3 h and 7 days after HI. Within the hypothermic group, T2 MRI scores were lower 7 days after HI than at 3 h (#*p* < 0.05, related samples Wilcoxon signed rank test). **(D)** The reduction in brain injury at 7 days in the hypothermic group was confirmed by histopathology (*p < 0.05, *t*-test).

Phase 3 was undertaken to determine whether the early neuroprotective effects of hypothermia observed at 3 h correspond with the MRIs and histopathology at 7 days (Figure 5). Within the normothermic treatment group, there was no significant difference in T2 MRI scores at 3 h and 7 days (*p* = 0.21*)*. However, in the hypothermic group, T2 MRI brain injury scores were lower 7 days after HI than 3 h after HI (related-samples Wilcoxon signed-rank test, *p* < 0.05), suggesting that hypothermic neuroprotection continued to evolve after the initial imaging at 3 h. With the smaller number of subjects used in this study (7 or 8 per treatment group), hypothermic pups showed a trend for lower MRI scores compared to normothermic pups at 3 h (Mann–Whitney *U*-test, *p* = 0.072), and this difference became significant at 7 days (Mann–Whitney *U*-test, *p* < 0.05). The histopathological analysis also showed less brain injury in hypothermic subjects than in normothermic subjects, 7 days after HI (*t*-test, *p* < 0.05). Refer to Table 1 for number of animals used.

**Figure 6:**
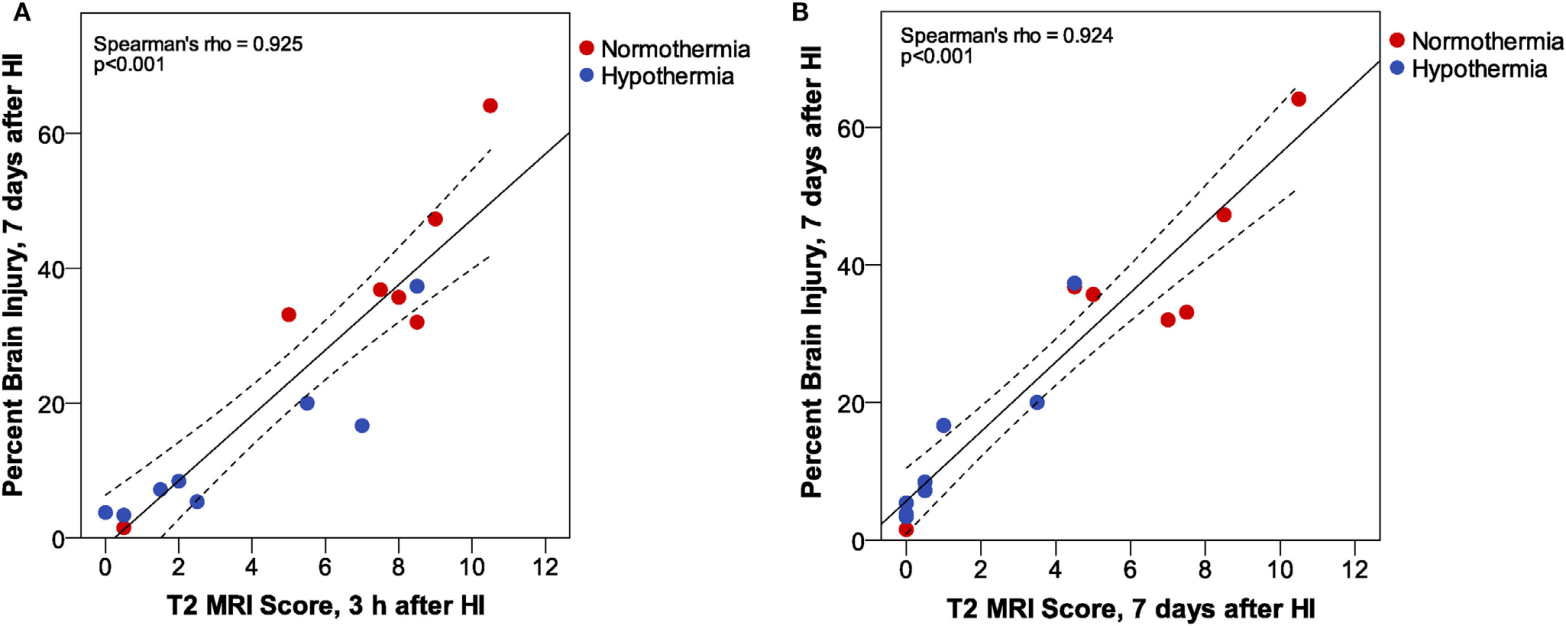
Phase 3: T2 magnetic resonance imaging (MRI) scores at **(A)** 3 h or **(B)** 7 days after hypoxic ischemia (HI). At both MRI time points, we found a very strong correlation between MRI score and histopathologic brain injury at 7 days. However, inspection of the correlation plots reveals less sensitivity for detection of subtle injury in MRI scores at 7 days. MRI scores are generally lower at 7 days than at 3 h after HI, and the subtle atrophy that is detected by quantitative histopathology is not readily apparent in T2 MRI at 7 days. (Dashed lines show 95% confidence interval for the mean.)

T2 MRI scores at 3 h and 7 days after HI in Phase 3 were compared with histopathological assessment of percent brain injury 7 days after HI by correlation analysis (Figure 6). These results confirm the very strong correlation between T2 MRI, at 3 h or 7 days after HI, with histopathological assessment of brain injury 7 days after HI. There is a somewhat greater spread of scores, especially for the mildly injured cases, in T2 MRI at 3 h than at 7 days after injury. This may reflect the ease of detecting small patches of hyperintense signal in the early scans, compared to the difficulty in detecting subtle atrophy at 7 days.

## Discussion

The goal of our Phase 1 study was to determine if, in this mouse model of neonatal HIE, initial injury could be consistently detected in T2-weighted or diffusion-weighted MRI at a very early time point, just 3 h after HI. A secondary goal was to optimize imaging for efficient assessment of initial injury in 10–20 subjects within an imaging session, while limiting the amount of additional anesthesia and potential interference with thermal control. We found that both imaging methods detected brain injury with similar extent and distribution. However, T2-weighted imaging provided a more detailed assessment of the extent and pattern of injury, with less time required for imaging at higher resolution. Injury was scored in four regions that were readily delineated in the T2 images, using a scale similar to that used previously to score brain injury in histopathological sections (28). T2 MRI injury scores 3 h after HI were highly correlated with a volumetric assessment of histopathological brain injury examined 7 days after HI.

Previous studies in rats subjected to HI on P7 demonstrated that T2 MRI and DWI methods show transient changes in signal intensity during or immediately after HI that return toward normal values 1–3 h later, with a secondary increase 12–24 h after HI (21, 22). Nedelcu and colleagues compared T2 MRI and DWI with cellular histopathology, which showed that the early MRI changes correlate with neuronal cytotoxic edema, while the delayed MRI signal changes correlate with neuronal edema, glial activation and interstitial edema (22). DWI detects restrictions to water molecule diffusion, and reductions in the calculated ADC are thought to reflect cell swelling associated with cytotoxic edema, while increased T2 MRI signal is thought to reflect both cytotoxic and vasogenic or interstitial edema (30–33). In a neonatal rat stroke model, MRI signal changes were observed at earlier intervals after injury. Injured volumes in ADC maps obtained immediately after reperfusion correlated well (slope near 1.0) with histopathological outcome at 48 h, but overestimated histopathological injury 2–5 h after reperfusion. T2 maps accurately depicted the injured volume at this early interval [slopes 0.97 at 2 h and 1.04 at 4 h (27)]. The neonatal stroke model, in which permanent middle cerebral artery occlusion is combined with transient common carotid artery occlusion, produces a more focal lesion than HI, in which permanent common carotid artery occlusion is combined with a period of global hypoxia. Such differences between models may account for differences observed in the timing of MRI signal changes. Ådén and colleagues (23) examined T2 MRI and DWI in mice subjected to HI on P7 and found increased T2 values 3–6 h after HI and decreased ADC values 3 h after HI. Our data are similar, showing consistent increases in T2 signal at 3 h, with injury scores that are strongly correlated with brain histopathology at 7 days. Therefore, we chose T2 MRI for Phases 2 and 3, as it is faster, with minimal interruption to hypothermia and shorter periods of anesthesia for imaging, is less sensitive to motion, and offers higher resolution and better delineation of brain regions than DWI.

In a study of the effects of hypothermia on brain injury after HI in rats on P10, Patel and colleagues reported no hypothermic protection 24 h after HI but found delayed neuroprotection using T2 MRI at 2 weeks and histopathology at 12 weeks (34). Therefore, we did not expect to find a difference between treatment groups in T2 MRI 3 h after HI. However, we found significant early neuroprotection in the hypothermic group. We then confirmed that this early effect of hypothermia was sustained and provided even greater benefit when examined with T2 MRI and histopathology, 7 days after HI. Burnsed and colleagues (35) used T2 MRI to evaluate hypothermic neuroprotection 8 and 20 days after HI in immature C57Bl/6 mice. In their model, using a lower temperature for hypothermia (31°C) for a shorter period (4 h) than that used in the present study, hypothermia provided neuroprotection 8 days after HI but protection persisted only in males at 20 days. Further studies will be required to determine whether hypothermic neuroprotection persists at longer survival times in our model.

Therapeutic hypothermia may provide neuroprotection *via* multiple mechanisms. These include a reduction in brain energy utilization, normalization of protein synthesis, and regulation of microglial activity and cytokine production (36). In astrocytes, preservation of ATP may support the Na^+^ gradients that drive glutamate uptake (37), and changes in glutamate transporter gene expression may limit glutamate release from astrocytes (38). In an adult rat cardiac arrest model, post-ischemic hypothermia reduced striatal glutamate and dopamine release during reperfusion (39), and in an adult rat stroke model, intraischemic mild hypothermia blocked the increase in extracellular glutamate and reduced the release of dopamine after four-vessel occlusion (40). Thus, a reduction in neuronal release of neurotransmitters after HI may play a role in early hypothermic neuroprotection. In adult rats subjected to cardiac arrest and resuscitation, hypothermia reduced levels of reactive oxygen species and activation of caspase-3 in the hippocampus, 12–24 h after arrest (41). Such changes in early excitotoxic mechanisms may underlie the very early neuroprotective effects of hypothermia observed here. Hypothermic neuroprotection in human infants requires that hypothermia be initiated during the latent phase, within 6 h of birth asphyxia (10, 42). Cooling within 3 h of birth provides better psychomotor outcomes than cooling initiated 3–6 h after birth (43), supporting the hypothesis that this therapy has important effects on early mechanisms of injury. An 1H-MR spectroscopy study in infants with HIE treated with hypothermia reported decreased glutamate and aspartate levels in the brain and improved energy homeostasis during hypothermia, compared to after rewarming, which supports an early effect on excitotoxic mechanisms (44). Further studies will be needed to evaluate the effect of hypothermia on other early HI injury mechanisms such as astrocyte glutamate uptake and on cellular and interstitial edema.

The neuroprotection observed in Phase 3 of this study using T2 MRI at 7 days after HI was significantly greater than the initial protection observed at 3 h. This suggests that the additional 3 h of hypothermia after imaging at 3 h may improve neuroprotection, and/or that some of the neuroprotective effects of hypothermia may involve later mechanisms of injury such as inflammation. In neonatal rat HIE models, hypothermia reduces caspase-3 activation, apoptosis, and necrosis examined 24 h after HI (45), modifies complement factor expression (46), and reduces IL1β levels (47). Later effects of hypothermia on cytokine levels and microglial activation have been reported in an adult murine stroke model (48), and a shift in microglial polarization toward an M2 phenotype at 24 h has been observed after hypothermia in an adult rat TBI model (49). In human infants with HIE treated with hypothermia, a reduction in serum levels of the pro-inflammatory cytokine IL-6 and an increase in the anti-inflammatory cytokine IL-10 have also been observed (50). Other studies have demonstrated that cooled infants with favorable outcomes had low or declining serum levels of pro-inflammatory cytokines over time, while those with persistently elevated cytokine levels had worse outcomes (51–53). Thus, a reduction in delayed mechanisms of injury such as inflammation may be responsible for the increased neuroprotection that we observed at 7 days.

A study in neonates evaluating early MRI, obtained during hypothermia, found that imaging on day 1 of life often underestimated the extent of injury. However, MRI obtained on days 2–3 detected injury (especially visible in DWI/ADC) that was highly predictive of the injury later on (18). Among the 43 asphyxiated newborns that were scanned, 60% had an injury seen on the early MRI that was persistent on the late MRI (days 10 and 30), while 40% did not have an injury on both early and late MRIs. The authors concluded that the presence of injury detected by MRI during hypothermia may help identify infants who would benefit from adjunct therapy, especially as the majority of patients with injury also had clinical evidence of severe encephalopathy. Several studies and sub-study analyses have demonstrated that therapeutic hypothermia decreases MRI lesions in infants (17, 54). Our current study, in which we demonstrate an early response to hypothermia following HI, has translational relevance, because evaluating the response to hypothermia at an early stage may help identify patients who will benefit from hypothermia alone vs. those who may need additional therapies.

This study demonstrates both early and delayed neuroprotective effects of hypothermia, using T2 MRI in a mouse model of HIE. Because the progression of injury is likely to vary in different brain injury models, different species, and at different ages, the optimal imaging modality and timing for imaging and interventions such as hypothermia will need to be determined for each model. In the P7 CD1 mouse model used for our studies, T2 MRI provides an effective and efficient method for assessing injury as early as 3 h after HI. The very early neuroprotective effects of hypothermia observed in this study suggest that hypothermia has a notable impact on early mechanisms of injury and support the initiation of hypothermic intervention as soon as possible after diagnosis of HIE.

## Ethics Statement

All animal procedures were carried out in accordance with the recommendations of the National Research Council Guide for the Care and Use of Experimental Animals (29) and were approved by the Institutional Animal Care and Use Committee of Johns Hopkins University.

## Author Contributions

MW, JZ, CN, AF, and MJ contributed to the conception and design of the study; CN and GD conducted experiments for Phase 1; MW, PC, SD, AG, and MG conducted experiments for Phases 2 and 3; SD and MW performed the statistical analysis; SD and AG scored MR images, collected histopathological data, and wrote the first draft of the manuscript; and MW, JZ, and SK wrote sections of the manuscript. All authors contributed to manuscript revision, read, and approved the submitted version.

## Conflict of Interest Statement

The authors declare that the research was conducted in the absence of any commercial or financial relationships that could be construed as a potential conflict of interest.

## Abbreviations

ADC, apparent diffusion constant; CP, cerebral palsy; DWI, diffusion-weighted MRI; EPI, echo planar imaging; FOV, field of view; HI, hypoxic ischemia; HIE, hypoxic ischemic encephalopathy; MRI, magnetic resonance imaging; P, postnatal day; RARE, rapid acquisition with relaxation enhanced; TE, echo time; TR, repetition time.
